# Social-Cognitive Network Connectivity in Preterm Children and Relations With Early Nutrition and Developmental Outcomes

**DOI:** 10.3389/fnsys.2022.812111

**Published:** 2022-04-07

**Authors:** Julie Sato, Marlee M. Vandewouw, Kristina Safar, Dawn V. Y. Ng, Nicole Bando, Deborah L. O’Connor, Sharon L. Unger, Elizabeth Pang, Margot J. Taylor

**Affiliations:** ^1^Diagnostic Imaging, Hospital for Sick Children, Toronto, ON, Canada; ^2^Department of Psychology, University of Toronto, Toronto, ON, Canada; ^3^Division of Neurosciences and Mental Health, Hospital for Sick Children, Toronto, ON, Canada; ^4^Autism Research Centre, Holland Bloorview Kids Rehabilitation Hospital, Bloorview Research Institute, Toronto, ON, Canada; ^5^Institute of Biomedical Engineering, University of Toronto, Toronto, ON, Canada; ^6^Division of Translational Medicine, The Hospital for Sick Children, Toronto, ON, Canada; ^7^Department of Nutritional Sciences, University of Toronto, Toronto, ON, Canada; ^8^Department of Paediatrics, Sinai Health, Toronto, ON, Canada; ^9^Department of Paediatrics, University of Toronto, Toronto, ON, Canada; ^10^Division of Neonatology, The Hospital for Sick Children, Toronto, ON, Canada; ^11^Division of Neurology, Hospital for Sick Children, Toronto, ON, Canada; ^12^Department of Medical Imaging, University of Toronto, Toronto, ON, Canada

**Keywords:** very low birth weight, preterm (birth), resting-state, MEG (magnetoencephalography), functional connectivity, social-cognition, outcomes, nutrition

## Abstract

Infants born very low birth weight (VLBW, < 1,500 g) are at a heightened risk for structural brain abnormalities and social-cognitive deficits, which can impair behavioural functioning. Resting-state fMRI, reflecting a baseline level of brain activity and underlying social-cognitive processes, has also been reported to be altered in children born VLBW. Yet very little is known about the functional networks underlying social cognition using magnetoencephalography (MEG) and how it relates to neonatal factors and developmental outcomes. Thus, we investigated functional connectivity at rest in VLBW children and the associations with early nutrition and IQ and behavioural problems. We collected resting-state MEG recordings and measures of IQ and social-cognitive behaviour, as well as macronutrient/energy intakes during initial hospitalisation in 5-year-old children born VLBW (*n* = 37) compared to full-term (FT; *n* = 27) controls. We examined resting-state network differences controlling for sex and age at scan. Functional connectivity was estimated using the weighted phase lag index. Associations between functional connectivity with outcome measures and postnatal nutrition were also assessed using regression analyses. We found increased resting-state functional connectivity in VLBW compared to FT children in the gamma frequency band (65–80 Hz). This hyper-connected network was primarily anchored in frontal regions known to underlie social-cognitive functions such as emotional processing. In VLBW children, increased functional connectivity was related to higher IQ scores, while reduced connectivity was related to increased behavioural problems at 5 years of age. These within-group associations were found in the slower frequency bands of theta (4–7 Hz) and alpha (8–12 Hz), frequently linked to higher-order cognitive functions. We also found significant associations between macronutrient (protein and lipid) and energy intakes during the first postnatal month with functional connectivity at preschool-age, highlighting the long-term impacts of postnatal nutrition on preterm brain development. Our findings demonstrate that at preschool-age, VLBW children show altered resting-state connectivity despite IQ and behaviour being in the average range, possibly reflecting functional reorganisation of networks to support social-cognitive and behavioural functioning. Further, our results highlight an important role of early postnatal nutrition in the development of resting-state networks, which in turn may improve neurodevelopmental outcomes in this vulnerable population.

## Introduction

The high prevalence of preterm births in Canada and globally (Public Health Agency of Canada, 2008, 2017; Chawanpaiboon et al., 2019), as well as the continued incidence of neurodevelopmental impairments (Aarnoudse-Moens et al., 2011; Twilhaar et al., 2018), has led to an increased interest in understanding the underlying neural mechanisms in this vulnerable population. This is a critical area of research given the increasing survival rates of infants born very preterm (VPT, < 32 weeks GA) and very low birth weight (VLBW, < 1,500 g) over the last decades due to advances in neonatal care (Blencowe et al., 2012; Chawanpaiboon et al., 2019). Improved survival has meant that more children born very preterm are now reaching school-age and displaying a wide range of difficulties across cognitive and social-cognitive domains, including lower IQ scores, academic underachievement, hyperactivity and attention problems, and poorer social competence compared to their full-term (FT) peers (Neubauer et al., 2008; Delobel-Ayoub et al., 2009; Ritchie et al., 2015; Twilhaar et al., 2018). While these difficulties have been reported across development, they become most apparent at early school-age (Aarnoudse-Moens et al., 2011; Mangin et al., 2017), a period of dynamic brain maturation and social development. However, despite these widespread impairments, little is known about the underlying neurophysiological mechanisms, particularly in preterm children born VLBW.

Evidence of resting-state functional connectivity (i.e., the coordinated and spontaneous communication among brain regions in the absence of a cognitive task) has been studied using functional MRI (fMRI) (Biswal et al., 1995; Raichle, 2015), and shown to be altered in infants and children born preterm (Damaraju et al., 2010; Smyser et al., 2011; Wehrle et al., 2018). These task-free scans collected at rest reflect a baseline level of brain activity and cognitive capacity for task processing (Gusnard et al., 2001). Importantly, these resting-state networks develop *in utero* during the third trimester (van den Heuvel and Thomason, 2016; De Asis-Cruz et al., 2020), a time of rapid brain maturation that can be adversely impacted by preterm birth and early exposure the extra-uterine environment. This is supported by fMRI studies in early infancy showing that precursors of the default-mode network (DMN)—a key resting-state network associated with social-cognitive processes (Schilbach et al., 2008)—were present in FT infants, but not preterm infants at term-equivalent age (Smyser et al., 2010). Importantly, the authors interpreted their findings as evidence of less mature functional connections within this network compared to those in FT infants. However, Lee et al. (2013), found longitudinal development (birth to 4 years of age) of core resting-state networks similar in VPT to what is seen in FT infants and young children. In another fMRI study, VPT children and adolescents showed widespread alterations in functional connectivity, particularly in resting-state networks involved in higher-order cognitive functions, compared to term-born peers. For instance, functional connectivity was found to be stronger between the sensorimotor network and regions of the salience network in VPT children and adolescents. Thus, these and other reports suggest that functional brain connectivity is disrupted and/or altered in children born preterm, and that these alterations may contribute to cognitive and behavioural difficulties observed in this population.

Only a few studies have used magnetoencephalography (MEG) to investigate resting-state connectivity in children born VPT and associations with neonatal predictors and developmental outcomes. MEG is an ideal neuroimaging modality for use in young children due to it being non-invasive and quiet, in addition to its good spatial and excellent temporal resolution (Hämäläinen et al., 1993; Hari and Salmelin, 2012). MEG is a direct measure of neural activity and able to capture the fast and frequency-specific oscillations involved in the formation of large-scale functional networks (Baillet, 2017). A recent MEG study found greater connectivity in the theta frequency band (4–7 Hz) at rest in 8-year-old children born extremely preterm (< 28 weeks GA) compared to children born VPT and FT (Kozhemiako et al., 2019). Increased oscillatory synchrony in theta was anchored in frontal regions and was associated with poor cognitive and behavioural scores, as well as adverse neonatal factors [e.g., gestational age (GA) at birth, early illness severity]. In line with these findings, we reported altered oscillatory mechanisms involving theta and alpha (8–14 Hz) during a working memory task in 6-year-old children born VPT compared to FT controls (Sato et al., 2019), suggesting a vulnerability of slow-wave frequency bands in this population. Of the few MEG studies conducted in children born preterm, none have looked at possible links to early postnatal nutrition, an important neonatal predictor of later developmental outcomes (Anderson et al., 1999; Isaacs et al., 2010; Cormack et al., 2019).

Emerging evidence suggests that optimising early postnatal nutrition, particularly macronutrient and energy intake, is associated with improved brain maturation (Beauport et al., 2017; Schneider et al., 2018; Blesa et al., 2019). Our recent study found that higher protein, lipid, and energy intake during the first postnatal month was associated with improved white matter microstructure in 5-year-old VLBW children from an overlapping sample in the present study (Sato et al., 2021a). Consistent with this, Schneider et al. (2018) reported that energy and lipid intake during the first 2 weeks of life was significantly associated with increasing fractional anisotropy between birth and term-equivalent age in preterm infants. To our knowledge, only one study has investigated the relations between early macronutrient intake in VPT infants and later resting state connectivity at school-age (Duerden et al., 2021). Using fMRI, the authors found that higher protein intake during the first month of life positively predicted the connectivity strength between a subcortical and cortical resting-state network at 7 years of age, which in turn was correlated with higher processing speed scores. No study, however, has evaluated the relations between early postnatal nutrition and whole-brain functional connectivity using MEG in children born VLBW.

Thus, this study investigated how resting-state functional connectivity was associated with neurodevelopmental outcomes at preschool-age in VLBW children, and the impact of early macronutrient and energy intake (during initial hospitalisation) in the VLBW group. We used a data-driven approach to explore network differences between VLBW and FT children at 5 years of age, to date the youngest cohort of VLBW children scanned at rest using MEG. We hypothesised that VLBW children would show altered functional connectivity at rest compared to their FT peers, and that these differences would be most apparent in the slow-wave frequency bands of theta and/or alpha. In addition, we expected that between-group network differences would primarily involve frontal regions, areas implicated in higher-order social and cognitive functions and that have been shown to be atypically recruited in previous resting-state and task-based paradigms in preterm populations (Frye et al., 2010; Ye et al., 2015; Kozhemiako et al., 2019). Further, we expected that these functional connectivity differences would be associated with cognitive and behavioural outcomes, as well as early macronutrient intake.

## Materials and Methods

### Participants

Eighty-six participants were recruited for this study. Of those, 56 children were born VLBW and were recruited as part of a 5-year follow up (NCT02759809) to the GTA-DoMINO study (ISRCTN35317141) examining the effect of donor milk, in comparison to preterm formula, as a supplement to mother’s milk in VLBW infants (Unger et al., 2014; O’Connor et al., 2016). The larger cohort included 363 VLBW infants who participated in the initial trial between October 2010 and December 2012 from four tertiary neonatal intensive care units (NICUs) in Southern Ontario, Canada. Infants were included in the trial if they weighed < 1,500 g at birth, were recruited within 4 days of birth and if enteral (oral) feeding was expected to begin within the first week of life. Exclusion criteria for VLBW infants included serious chromosomal or congenital anomalies that could affect neurodevelopment, severe birth asphyxia, participation in another nutrition intervention, or probable transfer to an NICU where the study protocol could not be continued. Thirty full-term (FT; > 37 weeks GA) children at 5 years of age were also recruited through hospital advertisements and word-of-mouth. Exclusion criteria for the FT group included prematurity, or presence of a learning, language or developmental disability. The study protocol was approved by the Hospital for Sick Children (SickKids) research ethics board. Written informed consent was obtained by parents and verbal assent was given by all children in accordance with the Declaration of Helsinki.

### Clinical and Demographic Information

Demographic characteristics were collected for all participants including scan age, sex, birth weight, GA at birth, breastfeeding duration, and maternal education level. In the VLBW group, perinatal radiological measures of brain injury were also assessed at birth by a radiologist and at least two neonatologists. Presence of brain injury was defined by at least one of the following cranial ultrasound findings: white matter lesions, echodense intraparenchymal lesions, periventricular leukomalacia, porencephalic cysts and ventriculomegaly with or without intraventricular haemorrhage. In addition to this, neonatal morbidities were also collected in the VLBW group during initial NICU hospitalisation. These clinical characteristics included presence of chronic lung disease (oxygen support at 36 weeks corrected age), and necrotising enterocolitis (Modified Bell Staging Criteria ≥ II), late-onset sepsis (positive blood or cerebrospinal fluid culture at ≥ 5 postnatal days), and retinopathy of prematurity.

### Neuropsychological Assessments

Developmental assessments were completed outside the scanner for all children at 5 years of age. Intelligence quotient (IQ) was assessed using the Weschler Preschool and Primary Scale of Intelligence IV (WPPSI-IV) (Wechsler, 2012), using Canadian norms. IQ scores were standardised with a population mean of 100 and a standard deviation of 15. To assess each child’s behavioural functioning, parents completed the Behaviour Assessment for Children, Third Edition (BASC-3) (Reynolds and Kamphaus, 2015). The BASC-3 composite scores include four areas: externalising problems, internalising problems, adaptive skills and behavioural symptoms index. Standardised T-scores were calculated for each BASC-3 composite score with a population mean of 50 and a standard deviation of 10.

### Early Macronutrient Intake in Very Low Birth Weight Children

Macronutrient (protein, lipid and carbohydrate) and energy intakes during initial hospitalisation were prospectively recorded for VLBW infants as previously reported (Ng et al., 2017, 2018; Asbury et al., 2019). All daily parenteral and enteral nutrition were recorded for each VLBW infant to estimate their daily macronutrient (in grammes per kilogramme per day) and energy (kilocalories per kilogramme per day) intakes. Consistent with previous studies, mean macronutrient/energy intakes were averaged for postnatal days 9–29 since nutrient fortification and full enteral feeding is largely established during this time interval (Stoltz Sjöström et al., 2013), whereas the first postnatal week (days 1–8), largely comprises of parenteral feeding and fluid loss (i.e., diuresis).

### Data Acquisition

All children completed 5-min of eyes-open resting-state in the MEG scanner using a 151-channel CTF system (CTF MEG International Service Ltd., Coquitlam, BC, Canada) at SickKids hospital. To improve compliance in this young cohort of 5-year-old children, a movie paradigm called “Inscapes” was used (Vanderwal et al., 2015). Inscapes has been validated for use in MEG and children, showing reduced head motion and cleaner MEG signal compared to a fixation cross resting-state (Vandewouw et al., 2021). Children lay supine in the MEG with their eyes open watching the Inscapes video, featuring slowly moving abstract shapes and soft music. MEG data were recorded continuously (600 Hz sampling rate), an online 150 Hz anti-aliasing filter was applied, and a third-order spatial gradient was used to cancel out external noise (0–150 Hz recording bandpass for anti-aliasing). Prior to MEG acquisition, fiducial coils were placed at the nasion and the left and right pre-auricular points to record head location continuously. For subject-specific head models, T1-weighted MRIs were acquired for each participant using a 3T Siemens MAGNETOM PrismaFIT with 20-channel head and neck coil (3D MRPAGE sequence: TR/TE = 1,870/3.14 ms, FA = 9°, FOV = 192 × 240 × 256 mm, 0.8 mm isotropic voxels). For co-registration of images, radio-opaque markers were placed at the same locations as the MEG fiducial coils.

### Preprocessing and Source Reconstruction

MEG resting-state data were processed using the Fieldtrip software toolbox (20150908 release; Oostenveld et al., 2011). Data were bandpass filtered offline at 1–150 Hz with fourth order two-pass Butterworth filter, and a Fourier transform notch filter at 60 and 120 Hz was applied to attenuate line noise. The 5-min MEG recordings were divided into 10 s epochs. Epochs where head position was more than 5 mm from the recording median position were excluded. Independent component analysis was used to remove artefacts related to cardiac activity and eye movements (Oostenveld et al., 2011). In addition, segments of data exceeding a threshold of ± 2 pT were excluded. Participant data were included in subsequent analyses if they had a least 1 min of resting-state data (≥ 6 epochs). A single-shell head model was computed from each participant’s MRI and co-registered to a template (ICBM 152) brain (Fonov et al., 2011). The 90-region Automated Anatomical Labeling (AAL) atlas (Tzourio-Mazoyer et al., 2002) was then used for source reconstruction. This atlas has been widely used in functional neuroimaging studies, including paediatric studies (Ye et al., 2015; Mossad et al., 2020), and provides good coverage of cortical and sub-cortical brain regions. The 90 seeds of the AAL atlas were then warped from standard template space into corresponding locations in each participant’s MRI-space. A linearly constrained minimum variance beamformer (Van Veen et al., 1997) was used to reconstruct the neural time-series for the 90 seeds of the AAL atlas. Using a two-pass FIR filter, time-series data for each AAL region were filtered into five canonical frequency bands: theta (4–7 Hz), alpha (8–14 Hz), beta (15–29 Hz), low gamma (30–55 Hz) and gamma (65–80 Hz).

### Resting-State Functional Connectivity

The Hilbert transform was applied to the filtered time-series to compute the instantaneous phase values for each AAL region and frequency band. To measure functional connectivity between pairs of AAL regions, the weighted phase lag index (wPLI) (Vinck et al., 2011) was used. The wPLI measures the consistency of the lag between two source regions, while giving optimal weighting to 90-degree phase differences making it less susceptible to artificial synchrony. Thus, the wPLI measures the non-zero phase leads and lags, providing values between 0 (no phase locking or synchronisation) to 1 (full phase-locking or synchronisation) for all source regions. These values were then averaged across 10 s epochs, resulting in a (90 × 90) connectivity matrix for each participant and frequency band.

### Statistical Analyses

#### Participant Characteristics

To test for group differences (VLBW vs. FT) in participant demographics and neuropsychological measures chi-squared tests were used for categorical variables and two-tailed *t*-tests or Mann-Whitney *U*-tests were used for continuous variables (when Shapiro-Wilks tests indicated non-normality). Differences in mean head motion and the number of resting-state epochs were also analysed between groups. Descriptive statistics were performed using Statistica (version 7.0; Statsoft Inc., Tulsa, United States) and significance was held at *p* < 0.05 in all cases.

#### Network Analyses

To examine functional connectivity at the network level, the network-based statistic (NBS; Zalesky et al., 2010, 2012; The Mathworks Inc., 2016) was used. NBS is a non-parametric technique that identifies significant differences in network connectivity while controlling for family-wise error rate. For each analysis, NBS applies a *t*-test to the z-scored wPLI values at every connection of the 90 × 90 connectivity matrix, which results in a *t*-value for each connection. The resulting test statistics are thresholded by a primary network component threshold, chosen to produce a network with ∼1% of total connections (∼40 connections) remaining. Thus, only contiguously connected nodes that exceed the threshold (i.e., components) are subjected to permutation testing (*n* = 5,000); significance is then assigned at the network level (a FWER corrected *p*-value is ascribed). This conservative threshold was chosen to target strong network differences in between- and within-group analyses. In primary analyses, connectivity matrices were submitted to NBS to test for (a) differences between VLBW and FT groups using a *t*-test, as well as (b) differences between groups in the association between functional connectivity and outcome (e.g., IQ, BASC). In secondary analyses, (c) the within-group associations with outcome measures were investigated, as well as (d) the associations with early nutrient intake within the VLBW group. A regression analysis was used to investigate the associations with outcome measures and nutrient intake. Age at scan and sex were used as covariates in all analyses. For each analysis, the participants wPLI values for each connection were *z*-scored and the statistical test was applied. Node degree, which is the number of connections extending from a node (i.e., brain region), was used to identify network hubs. Finally, significant networks were visualised using the BrainNet Viewer Connectivity Toolbox (Xia et al., 2013).

## Results

### Participant Characteristics

Of the 56 VLBW participants, nine children did not have sufficient resting-state epochs, 5 were unable/refused to complete the MEG scan, and 5 were scanned under a different resting-state protocol (fixation cross) and were thus not included in subsequent analyses. Of the 30 FT children, one child did not have sufficient resting state epochs and two children were scanned under a different protocol. Thus, the final sample consisted of 37 VLBW (16 females; mean ± SD age: 5.9 ± 0.2 years) and 27 FT (15 females; mean age: 5.7 ± 0.4 years) children. Descriptive statistics of demographic information and neuropsychological measures are summarised in Table 1. Clinical characteristics for the VLBW group are summarised in Table 2. VLBW and FT groups did not differ significantly in the proportion of males and females [X^2^(1) = 0.95, *p* = 0.33]. There was, however, a significant difference in the age at scan [*t*(50) = 2.66, *p* = 0.01], despite the VLBW participants being only 2 months older than the FT participants on average. There was a significant difference in maternal education level between groups [X^2^(2) = 12.56, *p* = 0.002], with education levels being lower in the VLBW compared to the FT group. As expected, there was a significant difference in breastfeeding duration between groups [*t*(53) = −2.7, *p* = 0.01], with the VLBW group having a shorter duration of breastfeeding days. Head motion and number of resting state epochs were also compared between groups and there were no significant differences [*t*(62) = −1.38, *p* = 0.17 and *t*(62) = 1.07, *p* = 0.29, respectively]. VLBW children had significantly lower IQ scores compared to FT children [*t*(59) = −2.23, *p* = 0.03], despite both groups performing within the average range of IQ scores. There were no significant group differences on any on the composite measures of the BASC−3, a parent-report questionnaire used to identify adaptive and problem behaviours in children (*p* > 0.05).

**TABLE 1:** Demographic and neuropsychological measures in VLBW and FT children.

	VLBW group (*N* = 37)	FT group (*N* = 27)	*p*-value
Age at scan (years)	5.9 ± 0.2	5.7 ± 0.4	0.01
Sex (M:F)	16:21	15:12	0.33
Birth weight (grammes)	970 {840, 1,250}	3,311 {3,039, 3,715}	6.2 × 10^–10^
Birth gestational age (weeks)	27.7 {26.7, 29.1}	39.4 {39, 40}	6.2 × 10^–10^
Breastfeeding duration (days)	176 {77, 304}	455.5 {182, 638}	0.03
**Maternal education level**			
High School	10/37 (27.0%)	0/27 (0%)	
University or College	23/37 (58.2%)	15/27 (55.6%)	0.0005
Post-graduate training	4/37 (10.8%)	12/27 (44.4%)	
Full-scale IQ	102.0 ± 14.0	109.7 ± 11.9	0.03
**BASC-3 composite scores** ^†^			
Externalising problems	47 {44, 53}	45 {42, 51}	0.38
Internalising problems	54.1 ± 11.2	49.4 ± 8.8	0.12
Behavioural symptoms index	50 {43, 53}	44 {41, 50}	0.11
Adaptive skills	52.6 ± 8.2	56.3 ± 7.8	0.11
Mean head motion (mm)	2.1 {1.1, 2.6}	2.2 {1.4, 3.3}	0.13
Mean # of epochs	25 {20, 28}	20 {18, 27}	0.24

*Categorical variables were presented as frequency (percentage) and continuous variables as mean ± SD, or median {IQR}.^†^Higher BASC-3 composite t-scores reflect increased risk of behavioural problems, apart from the adaptive scales, where higher values indicate lower risk.*

**TABLE 2:** Clinical characteristics in VLBW children.

	No./Total (%)
Brain injury	6/37 (16%)
Chronic lung disease (CLD)	5/37 (14%)
Necrotising enterocolitis (NEC)	0/37 (0%)
Late-onset sepsis (LOS)	11/37 (30%)
Retinopathy of prematurity (ROP)	0/37 (0%)

In our VLBW cohort, the mean (SD) macronutrient intake (g/kg/day) for postnatal days 9–29 was 3.6 (0.5) for protein, 4.9 (1.1) for lipid and 11.9 (1.3) for carbohydrate. The mean energy intake (kcal/kg/day) for postnatal days 9–29 was 105.6 (14.9). Further, 24 (65%) VLBW children achieved enteral recommendations of protein (3.5 g/kg/day), 23 children (62%) achieved lipid recommendations (4.8 g/kg/day), 25 (68%) achieved carbohydrate recommendations (11.6 g/kg/day), and 19 (51%) achieved energy recommendations (110 kcal/kg/day) (Koletzko et al., 2014).

### Between-Group Network Analyses

**FIGURE 1:**
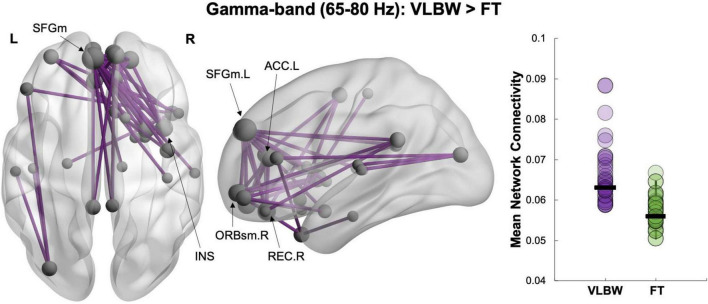
Between-group network analysis. Increased resting-state functional connectivity in VLBW compared to FT children in the gamma (65–80 Hz) frequency band (42 edges, 29 nodes, *p_*corr*_* = 0.03). Node size is scaled by degree (i.e., the number of connections a node has).

Our between-group contrast (controlling for sex and age at scan) revealed a network in the gamma (65–80 Hz) frequency band, in which VLBW children showed increased connectivity compared to FT controls (42 edges, 29 nodes, *p_*corr*_* = 0.03; Figure 1). This hyper-connected network was anchored in bilateral superior frontal (medial) and orbital frontal (bilateral medial and right superior) regions. In addition, the right insula, right rectus and the left anterior cingulate gyri were also major hubs in this network (see Supplementary Table 1 for a full list of network nodes). No significant group differences were found in the other frequency bands. There were also no differences between groups in the association between resting-state functional connectivity and outcome measures. Thus, associations between functional connectivity and the neuropsychological measures were examined across all subjects (VLBW and FT) and are reported in the following section “Associations With Neuropsychological Measures.” We also performed additional sensitivity analyses to examine the effect of breastfeeding duration and maternal education on our between-group effects. We found our between-group results were similar when controlling for breastfeeding duration (Supplementary Figure 2), such that VLBW children showed increased resting-state connectivity in the gamma-band compared to FT controls. In separate analyses, controlling for maternal education, we also found that results remained similar such that VLBW children showed increased gamma connectivity compared to controls (Supplementary Figure 3).

### Associations With Neuropsychological Measures

**FIGURE 2:**
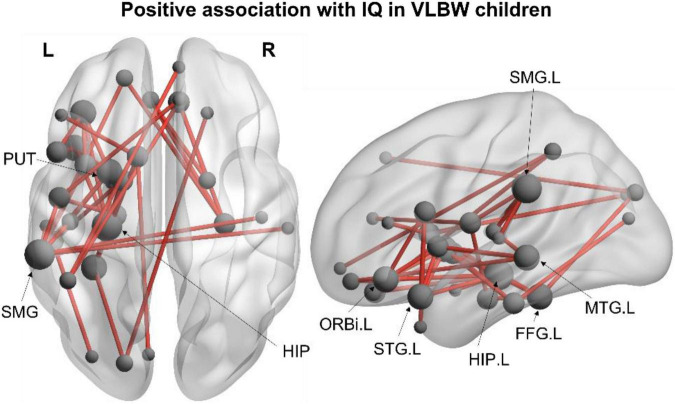
Within-group network analysis. Significant alpha-band (8–14 Hz) network positively associated with IQ in VLBW children (40 edges, 33 nodes, p*_*corr*_* = 0.033). Node size is scaled by degree.

The associations between functional connectivity and neuropsychological measures were also examined within each group. In the VLBW group, we found a network positively correlated with IQ in the alpha frequency band (40 edges, 33 nodes, *p_*corr*_* = 0.033; Figure 2), such that increased alpha connectivity within this network was associated with higher IQ scores. Increased connectivity was observed between frontal, temporal, subcortical and parietal regions in the left hemisphere, including the left hippocampus, supramarginal gyrus, inferior frontal gyrus (orbital), fusiform gyrus, putamen, superior temporal pole and middle temporal gyrus (see Supplementary Table 2 for a full list of network nodes). This association with IQ was not significant across all subjects, nor within the FT group.

**FIGURE 3:**
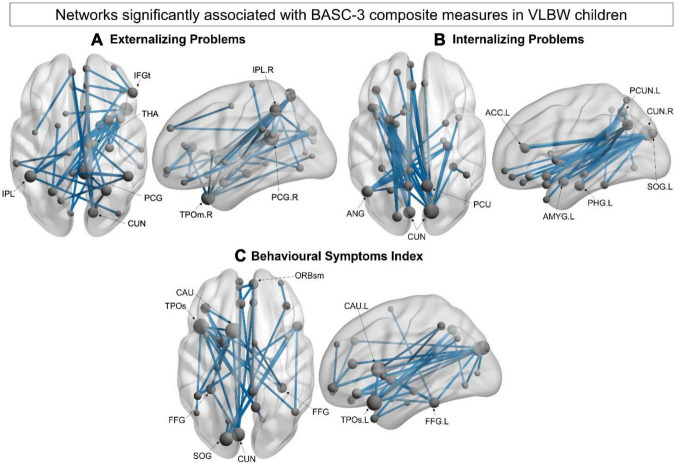
Within-group network analysis. **(A)** Theta-band (4–7 Hz) network significantly associated with BASC-3 externalising problems (44 edges, 36 nodes, *p_*corr*_* = 0.039) in VLBW children. **(B)** Alpha-band (8–14 Hz) network significantly associated with BASC-3 internalising problems (41 edges, 32 nodes, *p_*corr*_* < 0.001). **(C)** Alpha-band network significantly associated with the BASC-3 behavioural symptoms index (38 edges, 33 nodes, *p_*corr*_* = 0.002). All of these were negative correlations, such that reduced network connectivity was related to increased behavioural difficulties. Node size is scaled by degree.

Significant associations were also found with several BASC-3 composite measures in VLBW children. NBS results revealed a network in theta that was negatively correlated with the BASC-3 externalising problems composite measure (44 edges, 36 nodes, *p_*corr*_* = 0.039; Figure 3A). Higher BASC-3 composite scores reflect an increased risk of behavioural problems. Thus, lower connectivity within this network was correlated with more externalising problems. This theta-band network included bilateral parietal (superior and inferior) and right middle temporal (pole) regions, with other network hubs in the right posterior cingulate gyrus and subcortical regions (thalamus, putamen, and pallidum; Supplementary Table 3). A significant negative correlation was also observed between a network in alpha with the BASC-3 internalising composite measure (41 edges, 32 nodes, *p_*corr*_* < 0.001; Figure 3B). This alpha-band network was anchored in occipital (cuneus) and parietal (left angular and right praecuneus) hubs and included the left amygdala and parahippocampal gyrus (Supplementary Table 4). We also found a significant negative correlation between a network in alpha and the BASC-3 behavioural symptoms index (38 edges, 33 nodes, *p_*corr*_* = 0.002; Figure 3C) involving long-range connections between left occipital (superior and cuneus), superior temporal pole and subcortical (caudate) regions. This network also included hubs in the bilateral fusiform, left anterior cingulate, right posterior cingulate and right orbitofrontal (medial) gyri (Supplementary Table 5). No significant brain-behaviour associations were found in the FT group. However, across all subjects, we found similar network findings such that reduced functional connectivity was associated with worse behavioural problems in FT and VLBW children (see Supplementary Figure 1). In contrast to the within-group finding in VLBW children, we found two networks in alpha and gamma that were negatively correlated with externalising problems in all children. However, the network associations with internalising problems and behavioural symptoms index across both groups revealed very similar network distributions in alpha.

### Within-Group Associations With Early Nutrient Intake: Very Low Birth Weight Group

**FIGURE 4:**
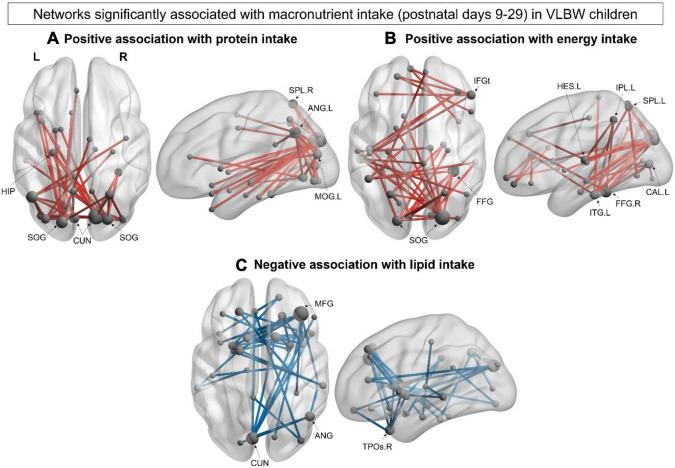
Within-group network analysis: VLBW group. **(A)** Significant alpha-band (8–14 Hz) network positively associated with higher protein intake (40 edges, 31 nodes, *p_*corr*_* = 0.032) in VLBW children. **(B)** Significant alpha-band (8–14 Hz) network positively associated with energy intake (40 edges, 36 nodes; *p_*corr*_* = 0.034). **(C)** Significant beta-band (15–29 Hz) negatively associated with lipid intake (41 edges, 37 nodes, *p_*corr*_* = 0.007). Node size is scaled by degree.

The association between early postnatal nutrition and functional connectivity at 5 years of age was also explored in the VLBW group. We found a significant positive correlation between mean protein intake during postnatal days 9–29 and a network in the alpha-band (40 edges, 31 nodes, *p_*corr*_* = 0.032; Figure 4A), such that greater protein intake was associated with increased connectivity within this network. This network was posteriorly anchored in occipital and parietal hubs, including the bilateral cuneus, bilateral superior occipital gyrus and left angular gyrus (Supplementary Table 6). A significant positive correlation was also found between mean energy intake during postnatal days 9–29 and a second alpha-band network (40 edges, 36 nodes; *p_*corr*_* = 0.034; Figure 4B). Similar to the first alpha-network, central hubs were found in occipital regions such as the right superior occipital and the left calcarine gyri, as well as the right fusiform, left inferior temporal and right inferior frontal (triangularis) gyri (Supplementary Table 7). Lastly, we found a significant negative correlation between mean lipid intake during postnatal days 9–29 and a network in the beta (15–29 Hz) frequency band (41 edges, 37 nodes, *p_*corr*_* = 0.007; Figure 4C), such that higher beta connectivity within this network was associated with lower lipid intake. This distributed beta-band network included hubs in the right middle frontal gyrus and left cuneus, with other network hubs in the right angular gyrus and middle temporal pole (Supplementary Table 8). We examined the association between functional connectivity and breastfeeding duration but found no significant associations.

We also performed a separate sensitivity analysis to examine the effect of neonatal morbidity (sepsis) on the association between nutrient intake and resting-state connectivity. We found similar results for the association between protein intake and resting-state connectivity, such that higher protein intake was associated with increased alpha connectivity (Supplementary Figure 3). However, the associations with lipid and energy intake were not significant when controlling for neonatal sepsis.

## Discussion

We investigated MEG resting-state functional connectivity in 5-year-old children born VLBW and relations with early macronutrient intake and developmental outcomes. We found that compared to FT controls, children born VLBW showed increased functional connectivity in gamma (65–80 Hz), a frequency band linked to a range of cognitive processes including attention (Fries, 2005), memory (Jokisch and Jensen, 2007) and perception (Melloni et al., 2007). We did not find any networks with significantly reduced functional connectivity in VLBW compared to FT children, nor any significant between-group differences on outcome measures. We did, however, find significant associations with IQ and behavioural problems in VLBW children. These significant associations were seen in theta- (4–7 Hz) and alpha-band (8–14 Hz) networks, frequencies implicated in long-range communication and commonly associated with learning and memory processes (Buzsáki and Draguhn, 2004; Palva et al., 2010). Within the VLBW group, we found that higher protein and energy intake during the first month of life (postnatal days 9–29) were associated with increased functional connectivity in the alpha-band, while higher lipid intake was associated with reduced beta-band (15–29 Hz) connectivity. These results are the first to establish frequency-specific connectivity patterns at the whole-brain level and their associations with early postnatal macronutrient intake in a cohort of VLBW children.

In our between-group contrast, we found hyper-connectivity during rest in children born VLBW compared to FT controls at preschool-age. Significant differences in connectivity were seen only in the gamma-band and in a network primarily anchored in frontal regions. These findings are consistent with previous structural and functional neuroimaging reports showing disrupted maturation of frontal regions following preterm birth (Taylor et al., 2012; Kozhemiako et al., 2019; Tokariev et al., 2019). This hyper-connected network included hubs in medial prefrontal areas (superior and orbitofrontal), as well as the right insula and left anterior cingulate gyrus. Importantly, these regions include core aspects of the DMN and the salience network—two resting-state networks that interact with each other to facilitate internally- and externally-directed cognitive processes (Seeley et al., 2007; Raichle, 2015). The DMN is involved in social cognition and active during social attribution tasks (i.e., attributing mental states or intentions to others), which have been shown to be impaired in preterm children born VLBW (Mars et al., 2012; Li et al., 2014; Williamson and Jakobson, 2014; Mossad et al., 2020). The salience network is involved in shifting attention and responding to salient stimuli and has also been reported to be active during emotional processing (Seeley et al., 2007; Uddin, 2015). Thus, the connections between brain regions implicated in navigating social situations, especially during the transitional preschool period, may be atypically recruited in VLBW children.

The only other MEG studies assessing source-level functional connectivity at rest in children born VPT have reported inconsistent results (Ye et al., 2015; Kozhemiako et al., 2019). However, this is likely due to the differing age-ranges of these study participants, as well as differences in sample size and methodologies. It has been widely reported that maturation and refinement of resting-state networks continues throughout childhood and adolescence (Fair et al., 2009; Hoff et al., 2013; Lee et al., 2013). The recent MEG study of Kozhemiako et al. (2019) found increased functional connectivity in theta in 8-year-old extremely preterm compared to VPT and FT children, which primarily involved frontal connections. The authors also reported increases in gamma connectivity in extremely preterm children, although these findings were less pronounced. Contrary to our hypothesis, we found hyper-connectivity in the gamma frequency band in 5-year-old children born VLBW. Oscillations in this frequency range have been linked to the maturation of cognitive functions (Engel et al., 2001; Uhlhaas et al., 2010) and are thought to arise via interactions between excitatory and inhibitory neurons in the cortex (Buzsáki and Wang, 2012). An imbalance of excitatory and inhibitory synaptic transmission may result from a disruption in the differentiation of cortical layers during the preterm period, leading to atypical recruitment of gamma oscillations (Kostović and Judas, 2002). However, these findings must also be considered within the context of the present VLBW cohort being studied, which were relatively high-functioning and without major intellectual or behavioural impairments. Increased gamma connectivity may thus reflect functional reorganisation of neural networks to support cognitive processes—an adaptive strategy to overcome early disruptions in synaptic development. Further, unlike previous resting-state MEG studies that found alterations in multiple frequency bands (Ye et al., 2015; Kozhemiako et al., 2019), we only found differences in the gamma-band, suggesting less physiological network disruption in the present younger and high functioning cohort.

We also found associations between resting-state functional connectivity with cognitive and behavioural outcomes in children born VLBW. Increased alpha connectivity was associated with improved IQ scores in the VLBW group. This left-lateralised network involved inferior frontal and temporal regions typically recruited during language and memory tasks—both of which contribute to IQ and overall academic performance (Aeschlimann et al., 2017; Flensborg-Madsen and Mortensen, 2019). Although no group differences were found in the alpha-band, these findings suggest that reduced alpha connectivity between regions in this network may contribute to lower IQ scores in children born VLBW. These findings are consistent with previous studies linking alpha oscillations with attention and working memory (Palva and Palva, 2007; Palva et al., 2010), suggesting an important role of these oscillations in the maturation of cognitive functions. No significant associations with IQ were found within the FT group, nor significant between-group interactions.

We also found significant relations with parent-reported measures of behaviour. Decreased theta connectivity was associated with more externalising problems in children born VLBW. The theta frequency band is thought to mediate thalamocortical interactions, which have been reported to be affected in children born preterm (McQuillen and Ferriero, 2005; Smyser et al., 2010). This is consistent with our network findings showing decreased connectivity between the thalamus and other subcortical and cortical structures. Interestingly, a recent MEG study found reduced theta-band connectivity involving the right thalamus during processing of angry faces in VPT children (Mossad et al., 2020). These findings suggest that deficits in emotional processing, particularly negative emotions, may partially explain the higher incidence of externalising problems such as hyperactivity in this population. Further, the lateralisation of this theta-band network is consistent with studies showing right-hemisphere dominance when processing negative emotions (Lanteaume et al., 2007; Sato et al., 2021b). Thus, difficulties processing and regulating negative emotions may put young children born VLBW at higher risk for externalising problems at preschool-age. We also found that decreased alpha connectivity was associated with more internalising problems and overall behavioural problems in children born VLBW. In addition, we found that these network associations were very similar across all children (VLBW and FT), such that reduced functional connectivity was associated with more behavioural problems. The only difference we found was that the correlation with externalising problems emerged in both alpha and gamma frequency bands when all children were combined in the same correlation analysis. These results suggest an important developmental role of resting-state functional connectivity, especially in the slow-wave frequency bands of theta and alpha, in the social and behavioural development in both VLBW and FT children.

Finally, ours is the first MEG study to investigate associations between early postnatal macronutrient intake and resting-state functional connectivity in children born VLBW. Only one other fMRI study has investigated these relationships in VPT children and found significant associations between protein intake during the first month of life and functional connectivity between the thalamus and anterior DMN at 7 years of age (Duerden et al., 2021). Our study extends these findings by showing, in whole-brain MEG analyses, that higher protein and energy intake during the first month of life were associated with increased connectivity in the alpha-frequency band, whereas higher lipid intake was associated with decreased beta-band connectivity. Importantly, the present results complement our previous structural connectivity findings that showed higher intakes of protein and energy during the same postnatal period (days 9–29) were associated with improved white matter microstructure at 5 years of age (Sato et al., 2021a). These findings suggest that early macronutrient and energy intakes contribute to the maturation of both functional and structural connectivity in the preterm brain.

We also found similarities in the distribution of the network associations for protein and energy intake, such that both networks were posteriorly anchored in occipital hubs. Significant results were also found in the alpha-frequency band, suggesting shared mechanisms by which protein and energy impact resting-state networks underlying cognitive and socio-cognitive functions. This is further supported by our previous diffusion MRI study that found significant associations between protein and energy intake with DTI metrics within many overlapping white matter tracts (Sato et al., 2021a). Early protein intake is essential for synaptogenesis and myelination, which undergoes critical development during the preterm period (Georgieff, 2007; Cormack et al., 2019). In our cohort, 65% of VLBW infants achieved enteral protein recommendations during postnatal days 9–29, while just over half (51%) achieved energy recommendations. Thus, increasing both protein and energy intake during the first postnatal month may be an important factor in improving developmental outcomes in preterm infants (Stephens et al., 2009; Isaacs et al., 2010). In addition, we also investigated how a neonatal morbidity (sepsis) influenced the association between postnatal macronutrient intake and resting-state connectivity at 5 years. We found that the positive association between protein intake and resting-state connectivity remained significant when controlling for neonatal morbidity, emphasising the importance of early protein intake for the development of resting-state networks in VLBW children. In contrast, the associations between lipid and energy intakes with resting-state connectivity were no longer significant when controlling for neonatal morbidity. Since major neonatal morbidity is associated with a reduced likelihood of achieving nutrient recommendations (Ng et al., 2018), it is important for future studies with a larger sample of VLBW children to confirm these findings.

While our study has many strengths, including being one of the few MEG studies to investigate resting-state functional connectivity in children born VLBW and the first to assess the associations with early postnatal macronutrient intake, there are some limitations to consider. Firstly, due to our sample size, our results may not be generalisable to all VLBW children, but nonetheless provides important information about the formation of resting-state networks following preterm birth. Secondly, we acknowledge that our FT group came from households with relatively high maternal education levels—an important indicator of socioeconomic status, which could bias our results. However, despite this difference in maternal education levels, IQ was still within the average-range for FT children included in our study. To further examine the impact of this on our results, we performed a sensitivity analysis where we included maternal education as a covariate and re-ran our between-group contrast. We found that our results remained similar such that VLBW children showed increased gamma connectivity compared to FT controls.

In summary, we found altered resting-state functional connectivity in children born VLBW at 5 years of age. These findings suggest that connections among brain regions involved in both cognitive and social-cognitive functions develop differently following preterm birth. However, given that this VLBW cohort was relatively high-functioning, and the majority were without a history of neonatal brain injury, our findings may reflect a reorganisation of resting-state networks to support cognitive processing. We also found significant associations between functional connectivity with developmental outcomes and early nutrient intake in children born VLBW. Increased functional connectivity was associated with higher IQ scores, while reduced connectivity within resting-state networks were related to increased behavioural difficulties. Disrupted development of these networks may thus explain some of the commonly reported neurodevelopmental deficits reported in preterm and VLBW populations. Further, early postnatal (days 9–29) protein, lipid and energy intake were associated with resting-state functional connectivity at preschool-age, demonstrating the long-term impacts of early nutrition on later brain maturation. While further studies with larger, longitudinal samples are needed to confirm the present findings, our results suggest that optimising early postnatal macronutrient and energy intake is a promising strategy to improve outcomes in this vulnerable population.

## Data Availability Statement

The datasets presented in this article are not readily available because the clinical and demographic data of this study cannot be made available in order to protect the privacy and confidentiality of our participants; we do not have consent from participant families to share their anonymised data, nor do we have permission from the research ethics boards of our participating hospitals. However, the neuroimaging data are available upon reasonable request to the senior author. Requests to access the datasets should be directed to MT, margot.taylor@sickkids.ca.

## Ethics Statement

The studies involving human participants were reviewed and approved by the Hospital for Sick Children (SickKids) research ethics board. Written informed consent to participate in this study was provided by the participants’ legal guardian/next of kin.

## Author Contributions

JS contributed to the conceptualisation and design of the study, as well as the statistical analysis and writing the first draft of the manuscript. MV and KS contributed to the statistical analysis. DN and NB involved in the data collection and organised the database. DO’C, SU, and MT contributed to the conceptualisation and design of the study, funding, and supervision of the project. All authors contributed to manuscript revision, read, and approved the submitted version.

## Conflict of Interest

The authors declare that the research was conducted in the absence of any commercial or financial relationships that could be construed as a potential conflict of interest.

## Publisher’s Note

All claims expressed in this article are solely those of the authors and do not necessarily represent those of their affiliated organizations, or those of the publisher, the editors and the reviewers. Any product that may be evaluated in this article, or claim that may be made by its manufacturer, is not guaranteed or endorsed by the publisher.
