# Supplementary material for: Social-Cognitive Network Connectivity in Preterm Children and Relations With Early Nutrition and Developmental Outcomes
Source: Front Syst Neurosci. 2022 Apr 7;16:812111. doi: 10.3389/fnsys.2022.812111 (PMC9022474; doi:10.3389/fnsys.2022.812111)
Supplement: Supplementary file 1 [file Data_Sheet_1.docx]

**Supplementary Materials**

**
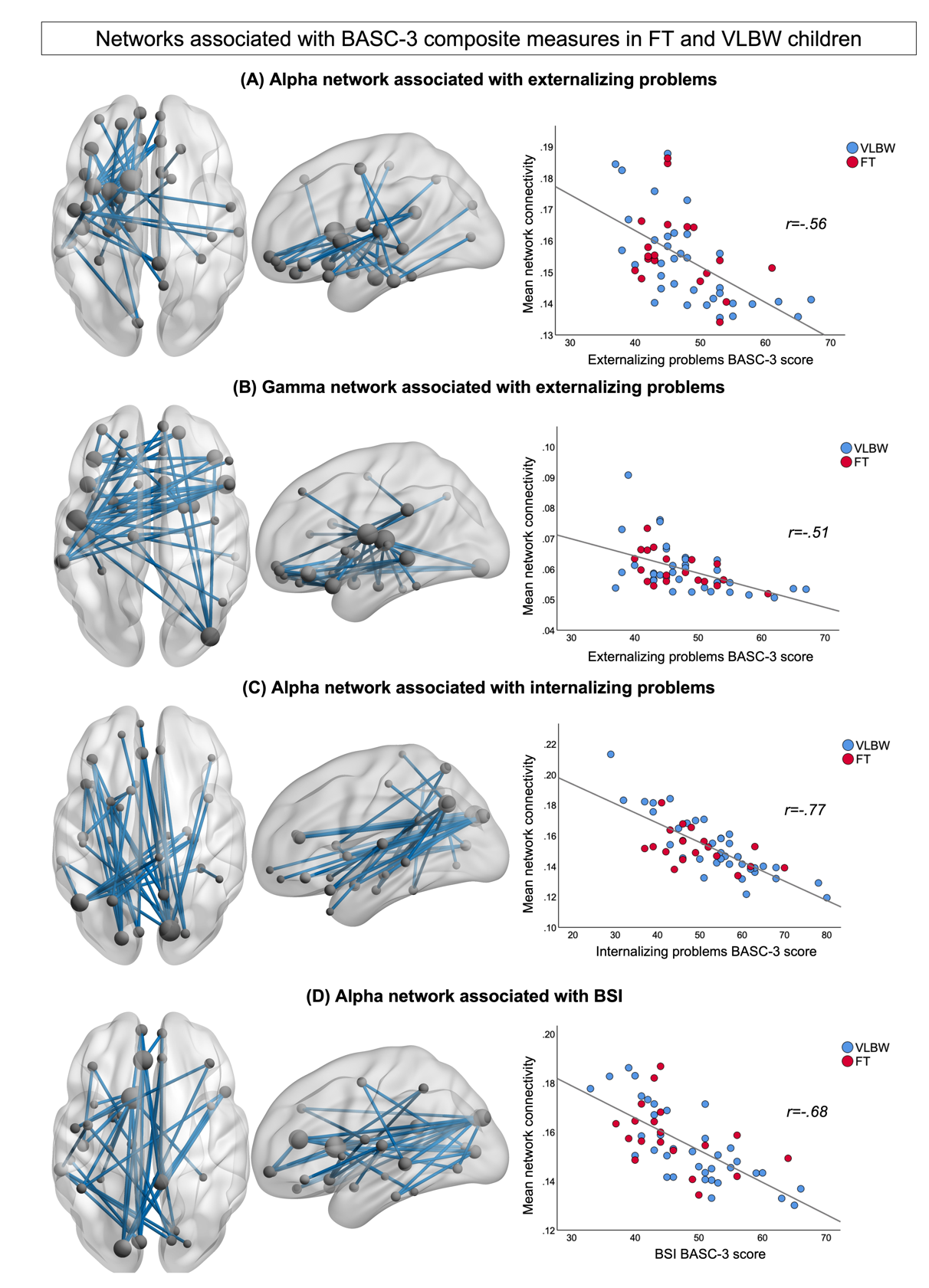
**

**Fig. 1.** Networks associated with BASC-3 composite measures in FT and VLBW children. (A) Alpha-band (8-14 Hz) network significantly associated with BASC-3 externalizing problems. (B) Gamma-band network (65-80 Hz) was significantly associated with externalizing problems. (C) Alpha-band networks negatively correlated with internalizing problems (C) and behavioural symptoms index of BASC-3 (D).


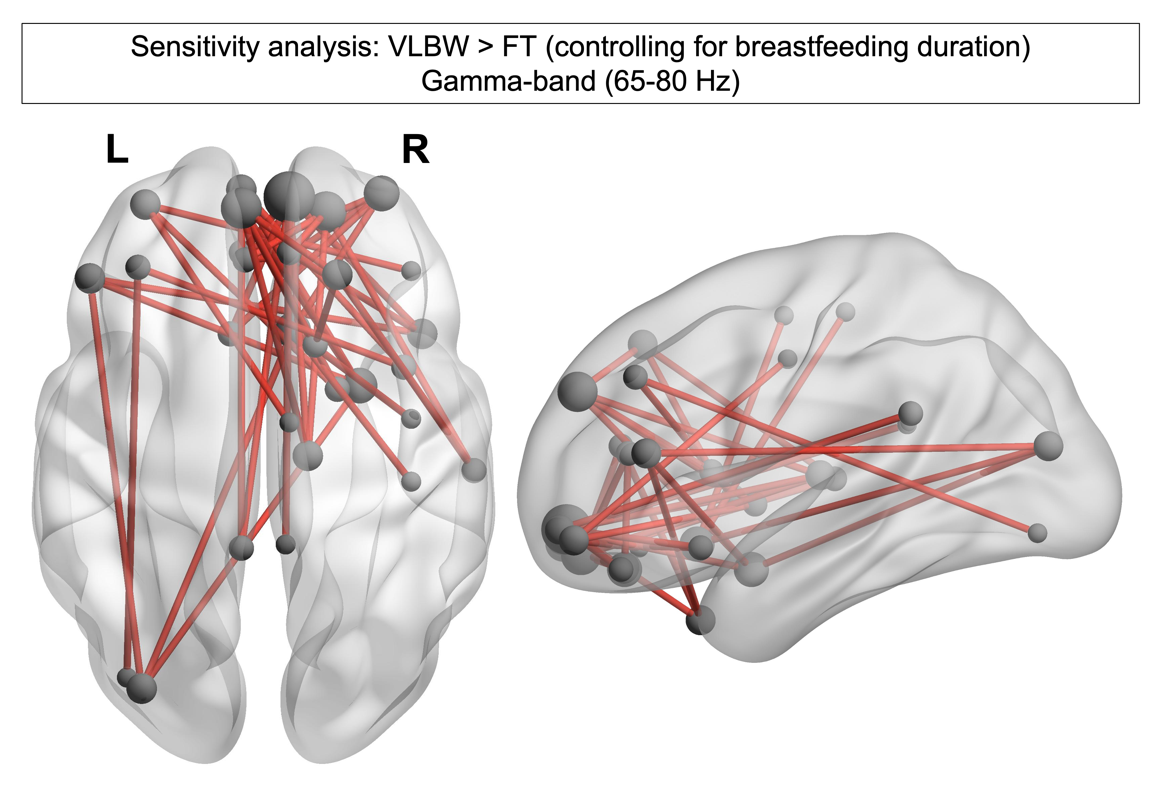


**Fig. 2**. Sensitivity analysis: Between-group network analysis (controlling for age, sex, and breastfeeding duration). Increased resting-state functional connectivity in VLBW compared to FT children in the gamma (65-80 Hz) frequency band (42 edges, 29 nodes, pcorr=0.031).


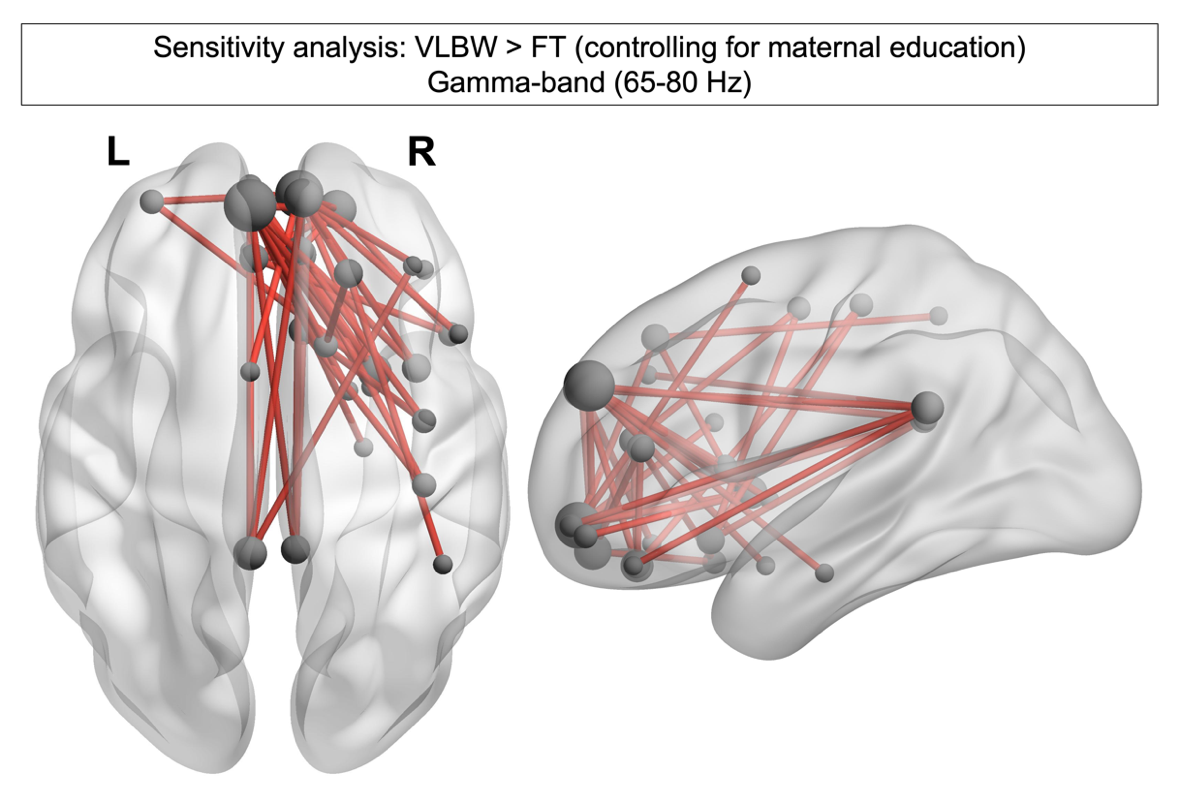


**Fig. 3**. Sensitivity analysis: Between-group network analysis (controlling for age, sex, and maternal education). Increased resting-state functional connectivity in VLBW compared to FT children in the gamma (65-80 Hz) frequency band (38 edges, 28 nodes, pcorr=0.046).


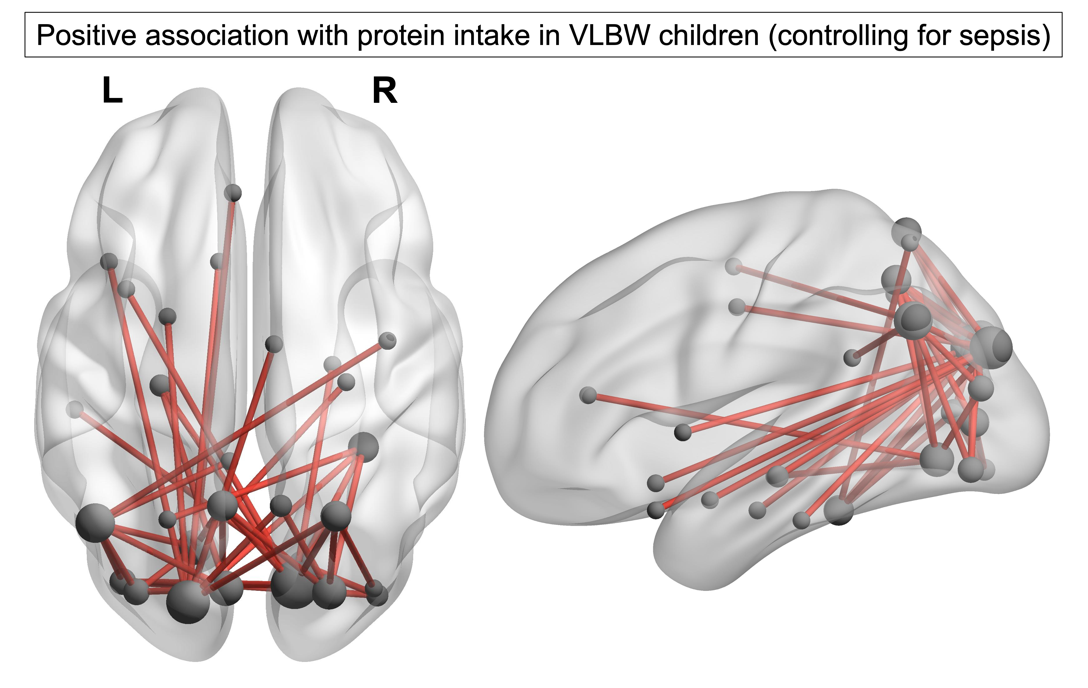


**Fig. 4**. Sensitivity analysis: Positive association between protein intake and resting-state connectivity in alpha-band (8-14 Hz) in VLBW children (40 edges, 29 nodes, *p_corr_*=0.035).

**Table 1.** AAL brain regions and associated number of connections involved in between-group gamma (65-80 Hz) network (42 edges, 29 nodes).

| **Brain regions (nodes)** | **Number of connections** |
| --- | --- |
| \| Precentral_R \| \| --- \| \| Frontal_Sup_Orb_R \| \| Frontal_Mid_Orb_L \| \| Frontal_Inf_Tri_L \| \| Olfactory_R \| \| Frontal_Sup_Medial_L \| \| Frontal_Sup_Medial_R \| \| Frontal_Med_Orb_L \| \| Frontal_Med_Orb_R \| \| Rectus_L \| \| Rectus_R \| \| Insula_R \| \| Cingulum_Ant_L \| \| Cingulum_Ant_R \| \| Cingulum_Post_L \| \| Cingulum_Post_R \| \| ParaHippocampal_L \| \| Amygdala_R \| \| Occipital_Mid_L \| \| Postcentral_R \| \| Caudate_R \| \| Putamen_R \| \| Pallidum_R \| \| Thalamus_R \| \| Heschl_L \| \| Temporal_Sup_L \| \| Temporal_Sup_R \| \| Temporal_Pole_Sup_R \| \| Temporal_Pole_Mid_R \| | \| 3 \| \| --- \| \| 5 \| \| 3 \| \| 2 \| \| 2 \| \| 11 \| \| 5 \| \| 4 \| \| 5 \| \| 1 \| \| 5 \| \| 5 \| \| 4 \| \| 3 \| \| 3 \| \| 2 \| \| 1 \| \| 2 \| \| 3 \| \| 1 \| \| 1 \| \| 4 \| \| 1 \| \| 1 \| \| 1 \| \| 1 \| \| 1 \| \| 1 \| \| 3 \| |

**Table 2.** AAL brain regions and associated number of connections involved in alpha-band (8-14 Hz) network positively associated with IQ in VLBW children (40 edges, 33 nodes).

| **Brain regions (nodes)** | **Number of connections** |
| --- | --- |
| \| Frontal_Sup_R \| \| --- \| \| Frontal_Sup_Orb_L \| \| Frontal_Inf_Oper_L \| \| Frontal_Inf_Tri_L \| \| Frontal_Inf_Orb_L \| \| Rolandic_Oper_L \| \| Frontal_Med_Orb_R \| \| Rectus_L \| \| Rectus_R \| \| Insula_L \| \| Cingulum_Ant_R \| \| Hippocampus_L \| \| Hippocampus_R \| \| ParaHippocampal_L \| \| Amygdala_L \| \| Cuneus_L \| \| Occipital_Sup_L \| \| Occipital_Mid_L \| \| Fusiform_L \| \| Parietal_Inf_L \| \| SupraMarginal_L \| \| Caudate_L \| \| Putamen_L \| \| Pallidum_L \| \| Pallidum_R \| \| Heschl_L \| \| Heschl_R \| \| Temporal_Sup_L \| \| Temporal_Sup_R \| \| Temporal_Pole_Sup_L \| \| Temporal_Mid_L \| \| Temporal_Pole_Mid_L \| \| Temporal_Inf_L \| | \| 1 \| \| --- \| \| 2 \| \| 3 \| \| 1 \| \| 4 \| \| 3 \| \| 1 \| \| 2 \| \| 3 \| \| 3 \| \| 1 \| \| 5 \| \| 3 \| \| 3 \| \| 1 \| \| 1 \| \| 2 \| \| 1 \| \| 4 \| \| 2 \| \| 5 \| \| 3 \| \| 4 \| \| 3 \| \| 2 \| \| 2 \| \| 1 \| \| 1 \| \| 1 \| \| 4 \| \| 4 \| \| 1 \| \| 3 \| |

**Table 3.** AAL brain regions and associated number of connections involved in theta-band (4-7 Hz) network associated with externalizing problems in VLBW children (44 edges, 36 nodes).

| **Brain regions (nodes)** | **Number of connections** |
| --- | --- |
| \| Precentral_L \| \| --- \| \| Frontal_Mid_Orb_R \| \| Frontal_Inf_Tri_R \| \| Frontal_Inf_Orb_R \| \| Olfactory_R \| \| Frontal_Sup_Medial_L \| \| Frontal_Med_Orb_R \| \| Rectus_L \| \| Rectus_R \| \| Insula_R \| \| Cingulum_Ant_L \| \| Cingulum_Ant_R \| \| Cingulum_Post_L \| \| Cingulum_Post_R \| \| Hippocampus_R \| \| Amygdala_R \| \| Calcarine_L \| \| Calcarine_R \| \| Cuneus_L \| \| Cuneus_R \| \| Lingual_L \| \| Occipital_Mid_R \| \| Parietal_Sup_L \| \| Parietal_Sup_R \| \| Parietal_Inf_L \| \| Parietal_Inf_R \| \| SupraMarginal_L \| \| Paracentral_Lobule_L \| \| Caudate_L \| \| Putamen_R \| \| Pallidum_L \| \| Pallidum_R \| \| Thalamus_R \| \| Temporal_Sup_R \| \| Temporal_Mid_R \| \| Temporal_Pole_Mid_R \| | \| 1 \| \| --- \| \| 1 \| \| 4 \| \| 2 \| \| 2 \| \| 2 \| \| 2 \| \| 1 \| \| 1 \| \| 3 \| \| 1 \| \| 1 \| \| 2 \| \| 7 \| \| 3 \| \| 3 \| \| 1 \| \| 1 \| \| 1 \| \| 4 \| \| 2 \| \| 1 \| \| 3 \| \| 4 \| \| 5 \| \| 4 \| \| 2 \| \| 1 \| \| 1 \| \| 4 \| \| 1 \| \| 4 \| \| 5 \| \| 1 \| \| 1 \| \| 6 \| |

**Table 4.** AAL brain regions and associated number of connections involved in alpha-band (8-14 Hz) network associated with internalizing problems in VLBW children (41 edges, 32 nodes).

| **Brain regions (nodes)** | **Number of connections** |
| --- | --- |
| \| Frontal_Sup_Orb_L \| \| --- \| \| Frontal_Inf_Orb_L \| \| Olfactory_L \| \| Frontal_Med_Orb_L \| \| Rectus_L \| \| Insula_L \| \| Insula_R \| \| Cingulum_Ant_L \| \| Cingulum_Ant_R \| \| Cingulum_Post_L \| \| Hippocampus_L \| \| Hippocampus_R \| \| ParaHippocampal_L \| \| ParaHippocampal_R \| \| Amygdala_L \| \| Amygdala_R \| \| Cuneus_L \| \| Cuneus_R \| \| Lingual_R \| \| Occipital_Sup_L \| \| Fusiform_L \| \| Fusiform_R \| \| Parietal_Sup_L \| \| Parietal_Inf_R \| \| Angular_L \| \| Precuneus_L \| \| Precuneus_R \| \| Caudate_L \| \| Putamen_L \| \| Pallidum_L \| \| Temporal_Pole_Sup_L \| \| Temporal_Pole_Mid_L \| | \| 1 \| \| --- \| \| 2 \| \| 3 \| \| 1 \| \| 1 \| \| 1 \| \| 1 \| \| 3 \| \| 1 \| \| 2 \| \| 1 \| \| 3 \| \| 4 \| \| 1 \| \| 4 \| \| 1 \| \| 6 \| \| 15 \| \| 1 \| \| 3 \| \| 1 \| \| 2 \| \| 1 \| \| 1 \| \| 5 \| \| 3 \| \| 6 \| \| 1 \| \| 3 \| \| 1 \| \| 2 \| \| 1 \| |

**Table 5.** AAL brain regions and associated number of connections involved in alpha-band (8-14 Hz) network associated with behavioural symptoms in VLBW children (38 edges, 33 nodes).

| **Brain regions (nodes)** | **Number of connections** |
| --- | --- |
| \| Frontal_Mid_R \| \| --- \| \| Frontal_Mid_Orb_R \| \| Frontal_Inf_Orb_L \| \| Supp_Motor_Area_R \| \| Olfactory_L \| \| Frontal_Sup_Medial_L \| \| Frontal_Med_Orb_L \| \| Frontal_Med_Orb_R \| \| Rectus_R \| \| Insula_L \| \| Insula_R \| \| Cingulum_Ant_L \| \| Cingulum_Post_R \| \| Hippocampus_L \| \| ParaHippocampal_R \| \| Amygdala_L \| \| Calcarine_L \| \| Cuneus_L \| \| Lingual_L \| \| Lingual_R \| \| Occipital_Sup_L \| \| Fusiform_L \| \| Fusiform_R \| \| Parietal_Inf_L \| \| Angular_L \| \| Angular_R \| \| Precuneus_R \| \| Caudate_L \| \| Putamen_L \| \| Pallidum_L \| \| Temporal_Pole_Sup_L \| \| Temporal_Pole_Sup_R \| \| Temporal_Pole_Mid_L \| | \| 2 \| \| --- \| \| 1 \| \| 3 \| \| 1 \| \| 1 \| \| 1 \| \| 2 \| \| 3 \| \| 2 \| \| 1 \| \| 1 \| \| 3 \| \| 3 \| \| 2 \| \| 1 \| \| 1 \| \| 1 \| \| 5 \| \| 1 \| \| 1 \| \| 5 \| \| 3 \| \| 3 \| \| 2 \| \| 1 \| \| 2 \| \| 3 \| \| 6 \| \| 1 \| \| 3 \| \| 6 \| \| 3 \| \| 2 \| |

**Table 6.** AAL brain regions and associated number of connections involved in alpha-band (8-14 Hz) network associated with protein intake in VLBW children (40 edges, 31 nodes).

| **Brain regions (nodes)** | **Number of connections** |
| --- | --- |
| \| Precentral_R \| \| --- \| \| Olfactory_L \| \| Insula_L \| \| Cingulum_Ant_L \| \| Cingulum_Mid_R \| \| Cingulum_Post_L \| \| Hippocampus_L \| \| Hippocampus_R \| \| ParaHippocampal_L \| \| Amygdala_L \| \| Calcarine_L \| \| Calcarine_R \| \| Cuneus_L \| \| Cuneus_R \| \| Lingual_L \| \| Occipital_Sup_L \| \| Occipital_Sup_R \| \| Occipital_Mid_L \| \| Occipital_Mid_R \| \| Occipital_Inf_L \| \| Occipital_Inf_R \| \| Fusiform_R \| \| Parietal_Sup_R \| \| Angular_L \| \| Precuneus_L \| \| Precuneus_R \| \| Pallidum_L \| \| Thalamus_R \| \| Temporal_Pole_Sup_L \| \| Temporal_Pole_Mid_L \| \| Temporal_Inf_L \| | \| 1 \| \| --- \| \| 1 \| \| 1 \| \| 1 \| \| 1 \| \| 1 \| \| 3 \| \| 1 \| \| 1 \| \| 1 \| \| 4 \| \| 3 \| \| 5 \| \| 9 \| \| 4 \| \| 6 \| \| 6 \| \| 4 \| \| 1 \| \| 3 \| \| 2 \| \| 3 \| \| 4 \| \| 6 \| \| 2 \| \| 1 \| \| 1 \| \| 1 \| \| 1 \| \| 1 \| \| 1 \| |

**Table 7.** AAL brain regions and associated number of connections involved in alpha-band (8-14 Hz) network associated with energy intake in VLBW children (40 edges, 36 nodes).

| **Brain regions (nodes)** | **Number of connections** |
| --- | --- |
| \| Frontal_Sup_Orb_L \| \| --- \| \| Frontal_Mid_R \| \| Frontal_Inf_Oper_R \| \| Frontal_Inf_Tri_R \| \| Frontal_Inf_Orb_L \| \| Frontal_Sup_Medial_R \| \| Frontal_Med_Orb_L \| \| Insula_R \| \| Hippocampus_L \| \| Hippocampus_R \| \| ParaHippocampal_R \| \| Calcarine_L \| \| Calcarine_R \| \| Cuneus_L \| \| Cuneus_R \| \| Lingual_L \| \| Lingual_R \| \| Occipital_Sup_L \| \| Occipital_Sup_R \| \| Occipital_Inf_R \| \| Fusiform_L \| \| Fusiform_R \| \| Postcentral_L \| \| Parietal_Sup_L \| \| Parietal_Sup_R \| \| Parietal_Inf_L \| \| Angular_R \| \| Paracentral_Lobule_R \| \| Caudate_L \| \| Pallidum_L \| \| Thalamus_L \| \| Thalamus_R \| \| Heschl_L \| \| Temporal_Sup_L \| \| Temporal_Inf_L \| \| Temporal_Inf_R \| | \| 2 \| \| --- \| \| 2 \| \| 1 \| \| 4 \| \| 1 \| \| 1 \| \| 2 \| \| 1 \| \| 2 \| \| 1 \| \| 2 \| \| 4 \| \| 1 \| \| 2 \| \| 2 \| \| 1 \| \| 2 \| \| 5 \| \| 9 \| \| 1 \| \| 2 \| \| 6 \| \| 1 \| \| 3 \| \| 2 \| \| 3 \| \| 1 \| \| 1 \| \| 1 \| \| 1 \| \| 1 \| \| 2 \| \| 3 \| \| 1 \| \| 4 \| \| 2 \| |

**Table 8.** AAL brain regions and associated number of connections involved in beta-band (15-29 Hz) network associated with energy intake in VLBW children (41 edges, 37 nodes).

| **Brain regions (nodes)** | **Number of connections** |
| --- | --- |
| \| Frontal_Sup_L \| \| --- \| \| Frontal_Sup_R \| \| Frontal_Sup_Orb_R \| \| Frontal_Mid_L \| \| Frontal_Mid_R \| \| Frontal_Inf_Tri_R \| \| Frontal_Inf_Orb_R \| \| Olfactory_L \| \| Olfactory_R \| \| Insula_L \| \| Insula_R \| \| Cingulum_Ant_L \| \| Cingulum_Post_L \| \| Hippocampus_R \| \| ParaHippocampal_R \| \| Amygdala_L \| \| Amygdala_R \| \| Calcarine_L \| \| Cuneus_L \| \| Cuneus_R \| \| Occipital_Sup_L \| \| Occipital_Inf_R \| \| Fusiform_R \| \| Postcentral_L \| \| SupraMarginal_R \| \| Angular_R \| \| Caudate_L \| \| Caudate_R \| \| Putamen_L \| \| Putamen_R \| \| Pallidum_L \| \| Thalamus_L \| \| Temporal_Pole_Sup_R \| \| Temporal_Mid_L \| \| Temporal_Pole_Mid_L \| \| Temporal_Pole_Mid_R \| \| Temporal_Inf_R \| | \| 3 \| \| --- \| \| 3 \| \| 2 \| \| 1 \| \| 8 \| \| 1 \| \| 2 \| \| 1 \| \| 2 \| \| 1 \| \| 3 \| \| 2 \| \| 1 \| \| 1 \| \| 2 \| \| 1 \| \| 1 \| \| 1 \| \| 6 \| \| 1 \| \| 1 \| \| 2 \| \| 1 \| \| 1 \| \| 2 \| \| 4 \| \| 3 \| \| 4 \| \| 3 \| \| 3 \| \| 4 \| \| 1 \| \| 1 \| \| 2 \| \| 1 \| \| 4 \| \| 2 \| |
